# Evidence for an expanded hypertension care cascade in low- and middle-income countries: a scoping review

**DOI:** 10.1186/s12913-022-08190-0

**Published:** 2022-06-27

**Authors:** Michael A. Peters, Caitlin M. Noonan, Krishna D. Rao, Anbrasi Edward, Olakunle O. Alonge

**Affiliations:** Department of International Health, Johns Hopkins Bloomberg School of Public Health, Baltimore, USA

**Keywords:** Hypertension, Hypertension management, Care cascade, Scoping review

## Abstract

**Background:**

With nearly 90% of annual hypertension-related deaths occurring in low- and middle-income countries (LMICs), there is an urgent need to measure the coverage of health services that effectively manage hypertension. However, there is little agreement on how to define effective coverage and the existing hypertension care cascade (hypertension prevalence, percent aware, percent treated, and percent controlled) does not account for the quality of care received by patients. This study reviews definitions of effective coverage and service quality for hypertension management services and proposes an expanded hypertension care cascade to improve measurement of health systems performance.

**Methods:**

A systematic scoping review of literature published in six electronic databases between January 2000 and October 2020 identified studies that defined effective coverage of hypertension management services or integrated dimensions of service quality into population-based estimates of hypertension management in LMICs. Findings informed an expanded hypertension care cascade from which quality-adjusted service coverage can be calculated to approximate effective coverage.

**Results:**

The review identified 18 relevant studies, including 6 that defined effective coverage for hypertension management services and 12 that reported a measure of service quality in a population-based study. Based on commonly reported barriers to hypertension management, new steps on the proposed expanded care cascade include (i) population screened, (ii) population linked to quality care, and (iii) population adhering to prescribed treatment.

**Conclusion:**

There is little consensus on the definition of effective coverage of hypertension management services, and most studies do not describe the quality of hypertension management services provided to populations. Incorporating aspects of service quality to the hypertension care cascade allows for the calculation of quality-adjusted coverage of relevant services, enabling an appropriate measurement of health systems performance through effective coverage.

**Supplementary Information:**

The online version contains supplementary material available at 10.1186/s12913-022-08190-0.

## Background

Hypertension, or raised blood pressure,^1^ is a leading cause of global cardiovascular mortality and morbidity, which causes one-third of all deaths globally [1]. Between 2000 and 2010, the age-standardized prevalence of hypertension fell by 2.6% in high-income countries, but rose by 7.7% in low- and middle-income countries (LMICs) [2]. In 2015, 8.5 million deaths were attributed to hypertension, 88% of which occurred in LMICs, underscoring the need for increased attention to hypertension management in these settings [3]. Hypertension can be controlled at the primary care level with a combination of sustained lifestyle changes and relatively affordable pharmaceuticals; however, successful treatment requires continuous monitoring and interaction with the health system. Successful management of hypertension at the population level is indicative of strong health system provision of preventive services. Therefore, measuring the coverage of hypertension management services that result in sustained non-elevated blood pressure levels can indicate health system performance.

Optimally organized health systems provide people with access to needed health services without causing financial hardship, but unless these services are provided at a certain standard of quality, they may not improve population health. It is widely accepted that expanding the coverage of health services alone is not sufficient to improve population health in maternal and child health interventions [4, 5]. This phenomenon has rarely been studied in services to address chronic diseases, such as hypertension. Without considering service quality, measurements of service coverage, also known as “crude coverage”, are only weakly associated with the health benefits received by a population [6]. Effective coverage is a promising metric for evaluating program and health system performance because it captures whether individuals are receiving health services of sufficient quality to achieve optimal health improvements made possible by medical and behavioral interventions [7]. For hypertension management, measuring and striving to increase effective coverage of services can improve early detection and initiation of treatment, ultimately reducing the burden of stroke and other consequences of high blood pressure.

Despite the promise of effective coverage and agreement on its basic calculation (*Effective Coverage* = *Utilization X Quality* | *Need*) there is not yet consensus on how to operationalize its measurement, especially when accounting for quality [8]. Quality of care has three aspects according to the Donabedian framework: structure (the inputs and resources needed to provide care), process (the actions taken by providers and patients in the act of giving and receiving care) and outcomes (the changes in patient health), each of which can be used to calculate effective coverage [9, 10]. An early article proposed six distinct approaches for calculating effective coverage, ranging from tracking changes in biomarkers over time to using statistical models to estimate health outcomes while accounting for unobserved variables such as intervention quality (Additional file 1) [7]. Previous studies have calculated effective coverage by adjusting crude intervention coverage levels according to a measures of intervention quality, such as service readiness observed, quality of care provided, or health outcomes achieved [5, 11–14]. Few of these studies have measured the effective coverage of interventions to manage hypertension or other non-communicable diseases (NCDs). More work is needed to incorporate relevant measures of quality into standard measures of effective coverage, especially for health conditions with a steadily increasing burden of disease, like hypertension.

National and sub-national efforts to calculate effective coverage of services primarily use health outcomes as a measure of intervention quality. While this approach has the benefit of providing an estimate of the health gains directly experienced by populations, many factors beyond the reach of the health system impact health outcomes. These factors, or social determinants of health, are interrelated non-medical factors such as early life exposures, social status, employment, social support and/or exclusion, and stress, all of which can influence health outcomes [15]. Measures of effective coverage that only adjust for quality based on health outcomes capture the impact of these social determinants and therefore may not reflect the direct contributions of health system performance in improving population health. Effective coverage based on non-elevated blood pressure levels is therefore also an indicator of broader societal factors, rather than health system performance alone. Methods for calculating effective coverage that consider the quality of services provided by the health system (i.e., structural and process quality) address this shortcoming.

Historically, population-level progress towards controlled blood pressure has been measured in a more or less standard way in the United States and internationally since at least the 1980s using a care cascade framework [16–18]. The care cascade usually involves measuring blood pressure levels of individuals identified through a population-based survey and reporting the following measures in a stepwise fashion:

(i) the prevalence of hypertension: the percent of population with elevated blood pressure readings on the day of the survey or reported using antihypertensive medicines,
(ii) the awareness of hypertension: the percent of those classified as hypertensive who had been previously diagnosed by a health worker,
(iii) the treatment of hypertension: the percent of hypertensives who report recently taking antihypertensive medicines, and
(iv) the control of hypertension: the percent of hypertensives who report taking antihypertensive medication and have non-elevated pressure on the day of the survey.

This standardized hypertension care cascade, measured at the population level, has enabled several powerful systematic reviews and meta-analyses on hypertension management nationally, regionally, and globally [1, 2, 19–21]. Applying the care cascade to different population subgroups enables important analyses on equity gaps in provision of care. Authors have also adapted the care cascade to meet their needs by including additional steps, including, for example, the screening of hypertension between steps i and ii [22, 23]. While the existing hypertension care cascade framework does incorporate a key measure of outcome quality (hypertension control), it does not account for the quality of health services that contribute to improved health. For other interventions, mostly related to maternal and child health, cascades of care have recognized this gap and have been expanded to measure process quality, in turn enabling the measurement of effective coverage [6, 12]. The absence of process quality-related indicators in the hypertension care cascade prohibits its ability to adequately measure health system performance related to hypertension care beyond the use of outcome indicators.

Without understanding the coverage of quality-adjusted services and examining relevant inputs and processes, health services research cannot reveal the drivers of and barriers to successful hypertension management and improved health. Thus, supply-side factors, such as facility readiness, provider knowledge and practices, and other health systems characteristics, should be considered and incorporated within the hypertension care cascade. This study seeks to review definitions of effective coverage for hypertension management services, including how non-outcome quality measures have been incorporated into the hypertension care cascade in studies relevant for low- and middle-income countries. Based on these findings, improvements to the care cascade framework will be proposed to inform improved measures of effective coverage of hypertension management services. A scoping study methodology was employed to accomplish this research aim, as it is a broader topic where many different study designs might be applicable [24, 25].

## Methods

The study followed Arksey and O’Malley’s process for conducting a scoping review, incorporating subsequent methodological advancements [24, 26]. One overarching research question was identified, specifically “how have measures of coverage of hypertension management services in LMICs considered aspects of service quality?”. Two sub-aims were identified, namely to describe how effective coverage of hypertension management has been defined and to describe how service quality has been incorporated into studies reporting hypertension cascades of care in LMICs. A review protocol is available upon request from the corresponding author.

To find relevant studies, we performed a search of electronic journals and databases including Scopus, EMBASE, PubMed, ScienceDirect, ProQuest, and Web of Science using keywords “hypertension” and “effective coverage” or “care cascade” and its variants. An additional search was conducted in a subset of databases to include published studies that included aspects of hypertension management (e.g. prevalence, treatment, and control) but did not mention the care cascade by name (see Additional file 2 for search strategy). The search strategy was calibrated to ensure that three pre-identified “tracer” articles that discussed effective coverage of hypertension management services were included in results [27–29]. These searches were conducted on 12 and 26 October 2020 and were supplemented by periodic searches of grey literature databases (Google Scholar, New York Academy of Medicine Library, and World Bank eLibrary) for additional information.

Identified data were collated, duplicate articles were removed, and titles and abstracts were screened for relevance in the Cochrane Community’s screening and data extraction tool, Covidence [30]. Relevant studies identified through title and abstract screening included those that (i) mentioned hypertension in the title, (ii) were conducted in a low- or middle-income country according to 2018 World Bank classifications, (iii) reported on data collected since 2000, (iv) were associated with a full-text manuscript in English (conference abstracts and commentaries were excluded but corresponding authors were contacted when possible), (v) used a population-representative study design (which is necessary to calculate coverage of a service in the general population), and (vi) reported sufficient information to calculate coverage. Any relevant study that mentioned “effective coverage” in the title or abstract was automatically included in the full-text review. Studies were excluded during the full-text review if they (i) did not report any measure of quality, (ii) reported on pregnancy-related hypertension, (iii) reported on specific populations (not age-related) that preclude generalization to entire populations, or (iv) included the phrase “effective coverage” but did not define the concept specifically for hypertension management services.

The full-text of selected studies was reviewed and relevant information (on study type, data sources, definition of effective coverage, incorporation of service quality, among others) was extracted in the online survey platform, Qualtrics [31]. At the title and abstract screening and full-text review stages, two reviewers examined each article, and conflicts were discussed and resolved by the lead author. Findings were summarized in tables that demonstrated (i) how “effective coverage” of hypertension management has been defined, and (ii) how dimensions of service quality have been incorporated into studies reporting population-level coverage of hypertension management services. For mixed methods studies, thematic synthesis was used to identify the largest relative challenges to providing quality care according to the Donabedian framework within qualitative results [32]. The quality of included articles was assessed using the Appraisal tool for Cross-Sectional Studies (AXIS) (Additional file 3) [33]. Findings were used to propose additional steps on the hypertension care cascade, including methods to improve the measurement of effective coverage for health systems performance evaluation, by comparing and aligning major gaps in effective coverage found in the literature with other models of expanded care cascades [4, 6, 12]. Finally, the revised framework was shared with a group of six experts which included cardiologists, providers with experience working in LMICs, and public health experts (on effective coverage and population-based hypertension management) purposively selected from among faculty and practitioners with affiliation with the primary academic institution where the research was based. Their oral feedback was collected over 3 review meetings which involved oral presentations by the first author, and further written feedback was collected via email communication. Feedback was incorporated to improve the overall framework.

## Results

### Findings from the scoping review

**Fig. 1:**
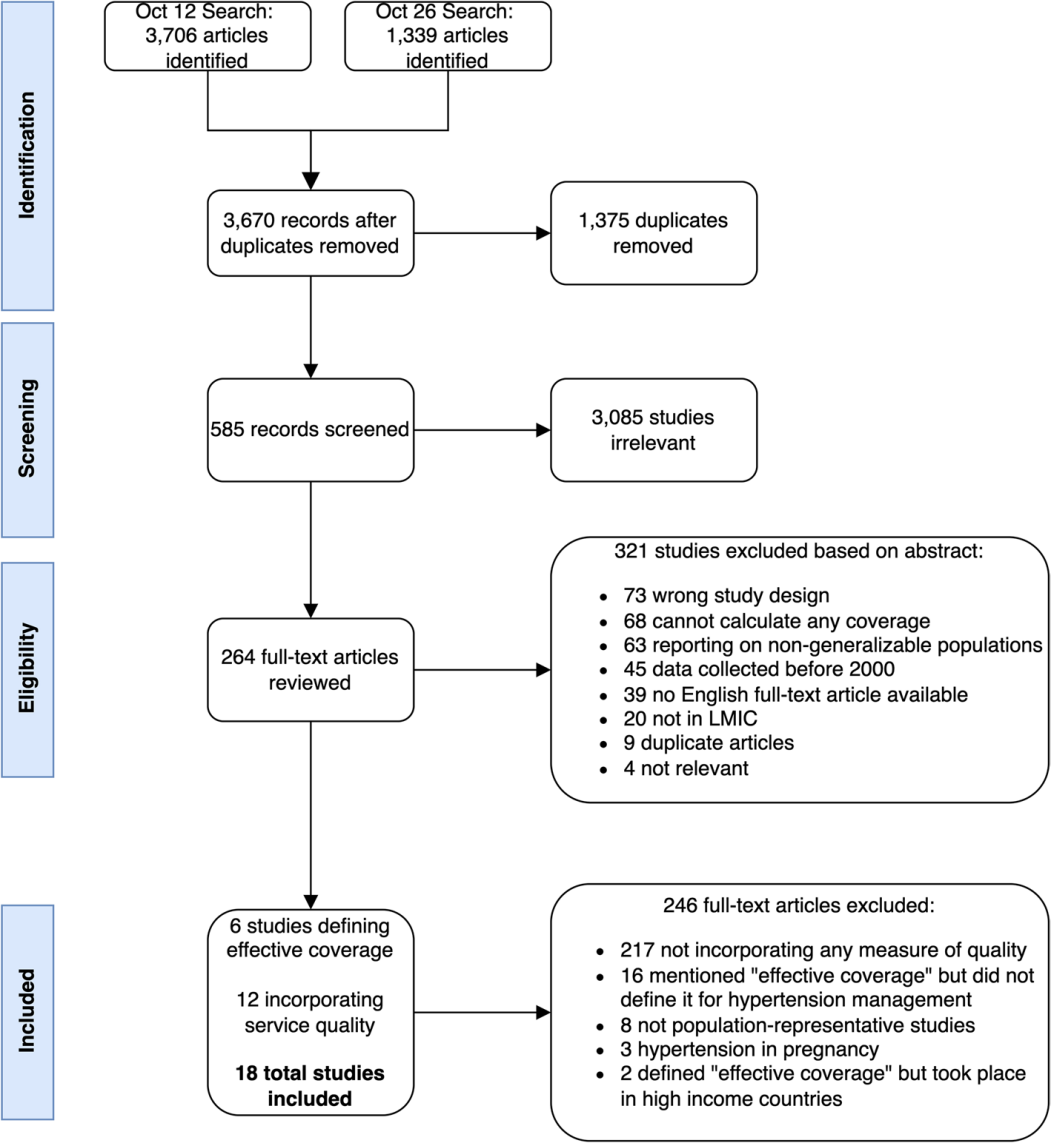
PRISMA flow diagram. PRISMA, Preferred Reporting Items for Systematic Reviews and Meta-Analysis

Across the databases, 5,045 records were identified, including 3,670 unique records that were screened for relevance. After title and abstract screening, 585 relevant records were assessed for eligibility for full-text review. Of these, 264 full-text records were reviewed, and 18 records were included that defined effective coverage of hypertension services (*n* = 6) or incorporated measures of service quality into their findings (*n* = 12) (Fig. 1). On the AXIS scale for rating the quality of observational studies, the 18 studies that informed the expanded hypertension care cascade were generally of high quality, scoring an average of 16.2 out of 20 (with scores ranging from 9 to 20).

**Fig. 2:**
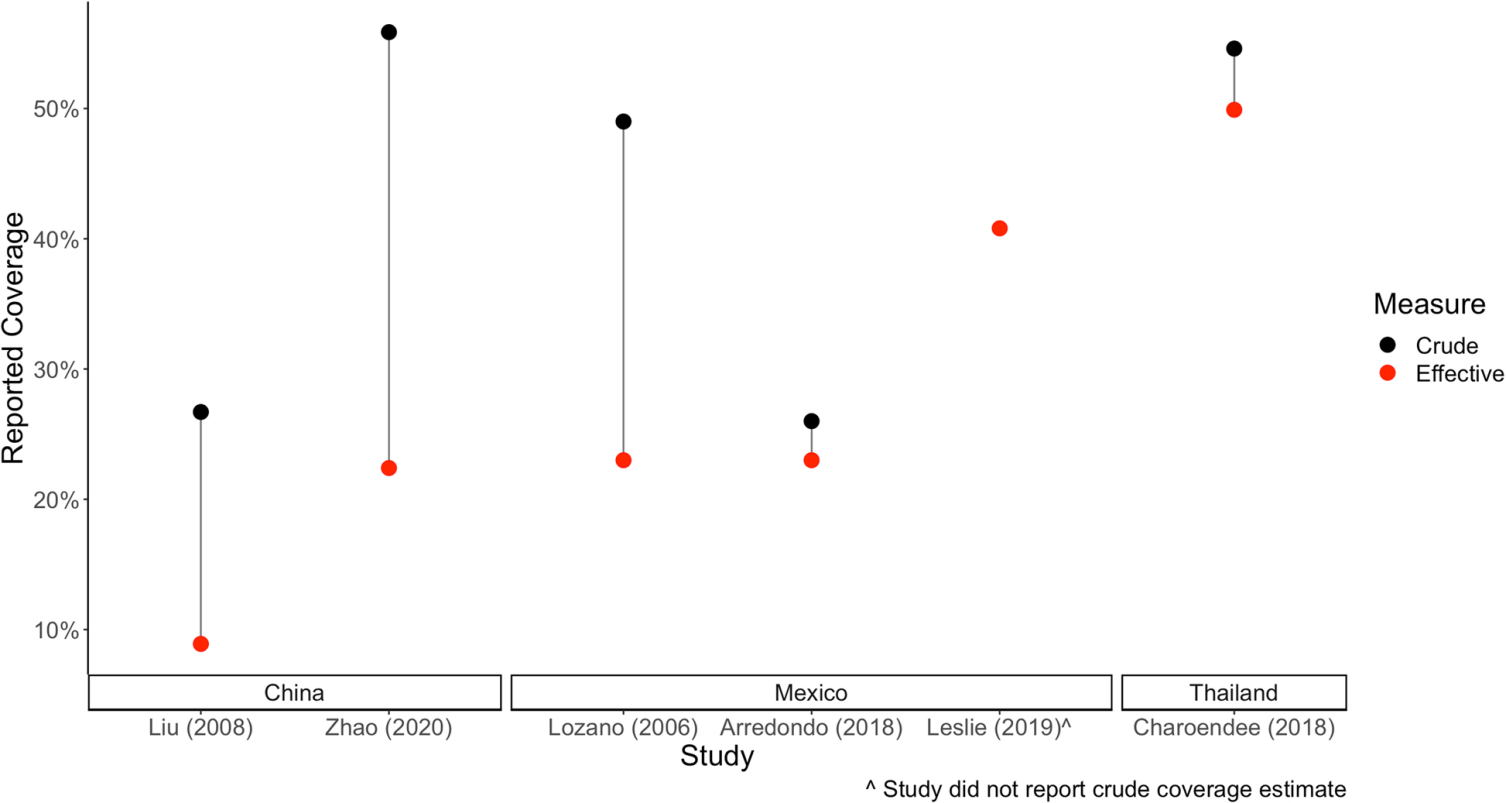
Reported differences in crude and effective coverage of hypertension management services

Studies that defined effective coverage took place in China, Mexico, and Thailand and were published between 2006 and 2020 (Table 1). Among the five studies that reported crude and effective coverage, the average difference in coverage estimate was 17.0% (Fig. 2). All six of the articles defining effective coverage reported some measure of outcome quality in their definition of effective coverage; however, there were differences in the way effective coverage was operationalized. Definitions of effective coverage included measures of actual reduction in blood pressure over target reduction [27, 34], the percent of the hypertensive population taking medication and achieving blood pressure control [28, 35], and the percent of the hypertensive population experiencing potential health gains (avoidance of hypertension-related hospitalization) [36]. One study considered a package of hypertension screening-related interventions and defined effective coverage for specific aspects of a national hypertension screening program [29]. Three of the studies defined effective coverage for hypertension management services in addition to other health services within the context of evaluating overall health systems performance [27, 28, 36]. Cross-sectional data sources were used in the majority of studies (5 out of 6), and longitudinal data sources were used in one study [34]. Out of the six studies, only one study reported any quality measure other than outcome quality. This study reported process quality indicators on the various screening-related services that were received by certain population segments in need [29].

**Table 1:** Definitions of effective coverage of services to measure hypertension

Author, year	Study Type/Data source	Study population	Definition of Effective Coverage	Quality Measure Reported		Effective vs Crude Coverage
Liu et al., 2008 [28]	Cross-sectional: 2004 China Adult Chronic Diseases Risk Factors Surveillance Survey	China, nationwide Adults age 18–69	The percentage of hypertensive people who reported having taken control measures and whose blood pressure was normal during the survey period	Outcome quality: Normal blood pressure during the survey period		Crude coverage: 26.7% Effective coverage: 8.9%
Zhao et al., 2020 [34]	Longitudinal: 2011 and 2013 China Health and Retirement Survey	China, nationwide Adults over age 45	The fraction of blood pressure reduction that is delivered to the population who take the anti-hypertensive medication	Outcome quality: Actual reduction in systolic blood pressure and/or diastolic blood pressure through taking antihypertensive medication from 2011 to 2013		Crude coverage: 55.9% Effective coverage: 22.4%
Lozano et al., 2006 [27]	Sequential cross-sectional: National survey in 2005–2006	Mexico, nationwide Adults over 20 years old	The ratio of actual reduction in systolic blood pressure to the difference between pretreatment systolic blood pressure and the target blood pressure for all individuals with hypertension (i.e., the proportion of the population reduction in blood pressure that can potentially be delivered through treatment that is actually delivered)	Outcome quality: Reduction in systolic blood pressure compared with treatment targets		Crude coverage: 49% Effective coverage: 23%
Arredondo et al., 2018 [37]	Sequential cross-sectional: Records of effective use of health services in 2005 and 2015	Mexico, selected states	The proportion of patients that effectively received care after demanding services to the health system for the control of hypertension	Outcome quality: Controlled blood pressure		Crude coverage: 26% Effective coverage:23%
Leslie et al., 2019 [36]	Cross-sectional: 2012 Mexican National Health and Nutrition Survey and national health information system	Mexico, nationwide	The proportion of individuals in need who experience potential health gains	Outcome quality: Blood pressure tests < 140/90 among patients with hypertension		Effective coverage: 40.8%
				Outcome quality: Patients with hypertension without hypertension-related hospitalization in past year		
Charoendee et al., 2018 [29]	Cross-sectional: Administrative data from outpatient services collected in 2013	Thailand, 76 provinces outside of Bangkok Population aged 15 years and older	The percent of population that receives appropriate hypertension screening and/or treatment based on their needs	Normotension	Process quality: Received at least one blood pressure measurement	Crude coverage: 54.6% Effective coverage: 49.9%
				Pre-hypertension	Process quality: Received hypertension and cardiovascular disease risk assessment	
				Suspected hypertension	Process quality: Received repeat blood pressure measurement within 2 months of initial screening	
					Process quality: Received cardiovascular disease risk assessment	
				Newly diagnosed hypertension	Process quality: Received early treatment	
					Outcome quality: Blood pressure lower than initial level or under control with serum lipid level better than initial test

The studies that considered coverage of hypertension services adjusted for aspects of service quality were published between 2007 and 2020 and took place across nine countries: Bangladesh, Brazil, Cuba, India, Kenya, South Africa, Tajikistan, Tanzania, and Uganda (Table 2). Only one study was representative at the national level [38]. Five studies incorporated measures of process quality, four studies incorporated measures of structural quality, and three studies described measures of both process and structural quality.

**Table 2:** Studies that incorporate service quality into measures of coverage

Author, year	Study Type/Data source	Study population	Care cascade	Quality Measure(s) Reported	Notes
Khanam et al., 2014 [39]	Cross-sectional: Household survey (no biomarkers)	Bangladesh: three rural sites (Matlab, Abhoynagar, and Mirsarai); Individuals aged 25 and above	Not explicitly defined	Process quality: Diagnosis by a qualified doctor, Adherence to treatment	Only about half of people with self-reported hypertension were diagnosed by qualified doctors; 26.2% of hypertensives were non-adherent to treatment
Macinko et al., 2018 [38]	Cross-sectional data: National Health Survey	Brazil, national; Adults 18 or older	Modified Cascade: Contact with the health system; Diagnosis; Receipt of treatment; Receipt of continuous, high-quality hypertension-related care; Blood pressure control and reduction of complications and/or physical limitations	Process quality: Continuous, high-quality care was defined as reporting no financial or organizational barriers to accessing hypertension-related healthcare, reporting that laboratory/diagnostic examinations were requested, that the provider knew about results of any diagnostics or lab-oratory exams (if requested), and receipt of all health advice	All quality measures are based on self-report
Londono et al., 2019 [40]	Cross-sectional: Household survey, health facility records	Cuba: two municipalities (Cardenas and Santiago); Hypertensive patients age 18 and older	Not explicitly defined	Process quality: Type of pharmacological treatment, Medication adherence	Used a linked survey study design; Receiving drugs and adherence were not associated with higher blood pressure control
Bhandari et al., 2015 [41]	Cross-sectional: Household survey	India: Urban slum dwellers in Kolkata; Hypertensive patients aged 25 and older	Standard Cascade: Prevalence of Isolated Systolic Hypertension; Awareness of Isolated Systolic Hypertension; Compliance to medication; Controlled blood pressure	Structural quality: Availability of medications	All quality measures are based on self-report; Patients adherent to prescribed medications were two times more likely to achieve blood pressure control than those who were not
				Process quality: Adherence to medication in the past week, Adherence to lifestyle modification advice (physical activity and salt restriction)	
				Outcome quality: Patient satisfaction	
Gabert et al., 2017 [42]	Mixed-methods (cross-sectional): Household and health facility surveys, focus group discussions, interviews	India: two districts (Shimla and Udaipur); Individuals aged 15 and above	Standard Cascade: Percent of hypertensives diagnosed; Percent of hypertensives receiving treatment; Percent of hypertensives with controlled blood pressure	Structural quality: Perceived lack of diagnostic equipment and testing capabilities (demand side) Patients were referred to private institutions or higher levels of care, stockouts were frequent, not enough time to counsel patients (supply side), Gaps in availability of diagnostic equipment and pharmaceutical supplies	Used a linked survey study design
Jayanna et al., 2019 [43]	Mixed-methods (cross-sectional): Household surveys, facility surveys, focus group discussions	India: one urban block in Mysore, Karnataka (population of 990,900); Adults over 18	Not explicitly defined	Structural quality: Facility readiness, human resources, availability of drugs	Used a linked survey study design to interview hypertensives identified in the first phase
				Process quality: Patient adherence to medicines	
Heller et al., 2020 [44]	Longitudinal: Household survey, health facility records	Kenya and Uganda: (32 communities, population of 157,985); Adults 18 or older	Modified Cascade: Adults enumerated; Adults attended Community Health Campaign; Attendees screened; Screened and hypertension-positive; Hypertension-positive and referred to care; Linked to care within two years; Patients retained after first visit; Blood pressure checked at last visit; Blood pressure normal at last visit	Process quality:Implementation fidelity of providers (e.g. asked history of hypertension, blood pressure checked twice, appropriate linkage to care, appropriate prescription based on examination); Retention in care (follow-up scheduled and attended, blood pressure checked)	Used a linked survey study design
Thorogood et al., 2007 [45]	Mixed-methods (cross-sectional): Household survey, rapid ethnographic assessment including interviews, focus groups, and participatory techniques	South Africa: one sub-district (Agincourt); Adults 35 or older	Not explicitly defined	Structural quality: Availability of drugs in clinics (stock outs), Clinics either had to deny treatment to patients or switch treatment to another drug- both were likely to reduce adherence, Lack of appropriate equipment	Hypertension management was studied in the context of the burden of stroke
Chukwuma et al., 2019 [46]	Mixed-methods (cross-sectional): Household surveys, facility registries, focus group discussions	Tajikistan: two regions (Sughd and Khatlon); Adults over 18	Modified cascade: Diagnosis; Treatment initiation; Treatment monitoring; Blood pressure control	Structural quality: Insufficient supply of equipment and human resources. Sphygmomanometers are not replaced and calibrated regularly	Also conducted a literature review on the range of clinical and non-clinical interventions that could overcome identified barriers These solutions included mobilizing faith-based organizations, scaling up screening through May Measurement Month and health caravans, leveraging service user interactions with pharmacy care, introducing job aids for providers, and task-shifting to increase provider supply
				Process quality: Current protocols lack clear guidance for each level of the health system	
Zack et al., 2016 [47]	Longitudinal: Household survey	Tanzania: peri-urban area near Dar es Salaam; Hypertensives 40 years or older	Standard Cascade: Percent of hypertensives diagnosed; Percent of hypertensives receiving treatment; Percent of hypertensives with controlled blood pressure	Process quality: Accessing health professional for follow up, Adherence to medication	All quality measures are based on self-report
Galson et al., 2017 [48]	Mixed-methods (cross-sectional): Household survey and focus group discussions and in-depth interviews with patients and providers	Tanzania: Kilimanjaro region; Adults 18 or older	Not explicitly defined	Structural quality: Long wait times, understaffing, lack of experience, and medication costs	A care cascade was not explicitly defined, but the study accounted for the type of treatment received by hypertensives (biomedicine or traditional medicine)
				Outcome quality: Perceived quality of biomedical healthcare delivery	
Wollum et al., 2018 [49]	Mixed-methods (cross-sectional): National household data, health facility surveys, focus group discussions, and key informant interviews	South Africa: two districts (Umgungundlovu and Pixley ka Seme) Adults 18 and over	Standard Cascade: Percent of hypertensives diagnosed; Percent of hypertensives receiving treatment; Percent of hypertensives with controlled blood pressure	Structural quality: Limited availability of testing equipment, Perceived prevalence of stockouts, Long wait times which reduced care-seeking and patient interest in returning for care	Used a linked survey study design

The most common study designs were mixed methods designs that paired quantitative population-based survey data with qualitative information collected from patients and/or providers. Mixed methods studies that incorporated qualitative data from patients described structural quality issues such long wait times, lack of drugs, and poor adherence, and outcome quality issues related to patient satisfaction (e.g. poor perceived quality of services) [46, 48, 49]. Providers described a lack of appropriate equipment, stockouts of medicines, and insufficient time to counsel patients on lifestyle advice [42, 45]. Studies that used linked population-based survey study designs with information collected in facilities were able to provide quantitative estimates about these structural quality constraints. Cross-sectional household surveys were also frequently used to understand additional information about hypertension treatment, primarily focusing on availability of health services (including screening and diagnosis), the specific types of medication taken, adherence to treatment, and patient satisfaction.

Although five studies did not explicitly define a care cascade and four studies used steps from the standard hypertension care cascade, three recently published studies proposed alternative hypertension care cascades. One study separated treatment into service initiation and continued treatment [46]. Another included supply-side considerations, namely contact with the health system (service availability) and the receipt of continuous, high-quality treatment (quality-adjusted treatment) [38]. The third study linked detailed information from a hypertension screening and management intervention with a household survey, included multiple steps related to screening, referral to care, linkage to care within two years, retention in care, and then characteristics about the care provided during provider interactions [44]. Of note, a series of World Bank reports on cascades of care for hypertension that identified supply- and demand-side bottlenecks to achieving hypertension control were identified through grey literature searches, which are presented as a supplementary table (Additional file 4).

The structure- and process-related quality features identified in the articles fall into three main categories: facility readiness (related to structural quality including equipment, medicines, and human resources), content of care (related to process quality including adherence to treatment protocols, type of pharmacological treatment prescribed, and health advice given), and patient adherence to treatment (relating to process quality including adherence to medicines and retention in care).

### Proposal for an expanded cascade of hypertension care

**Fig. 3:**
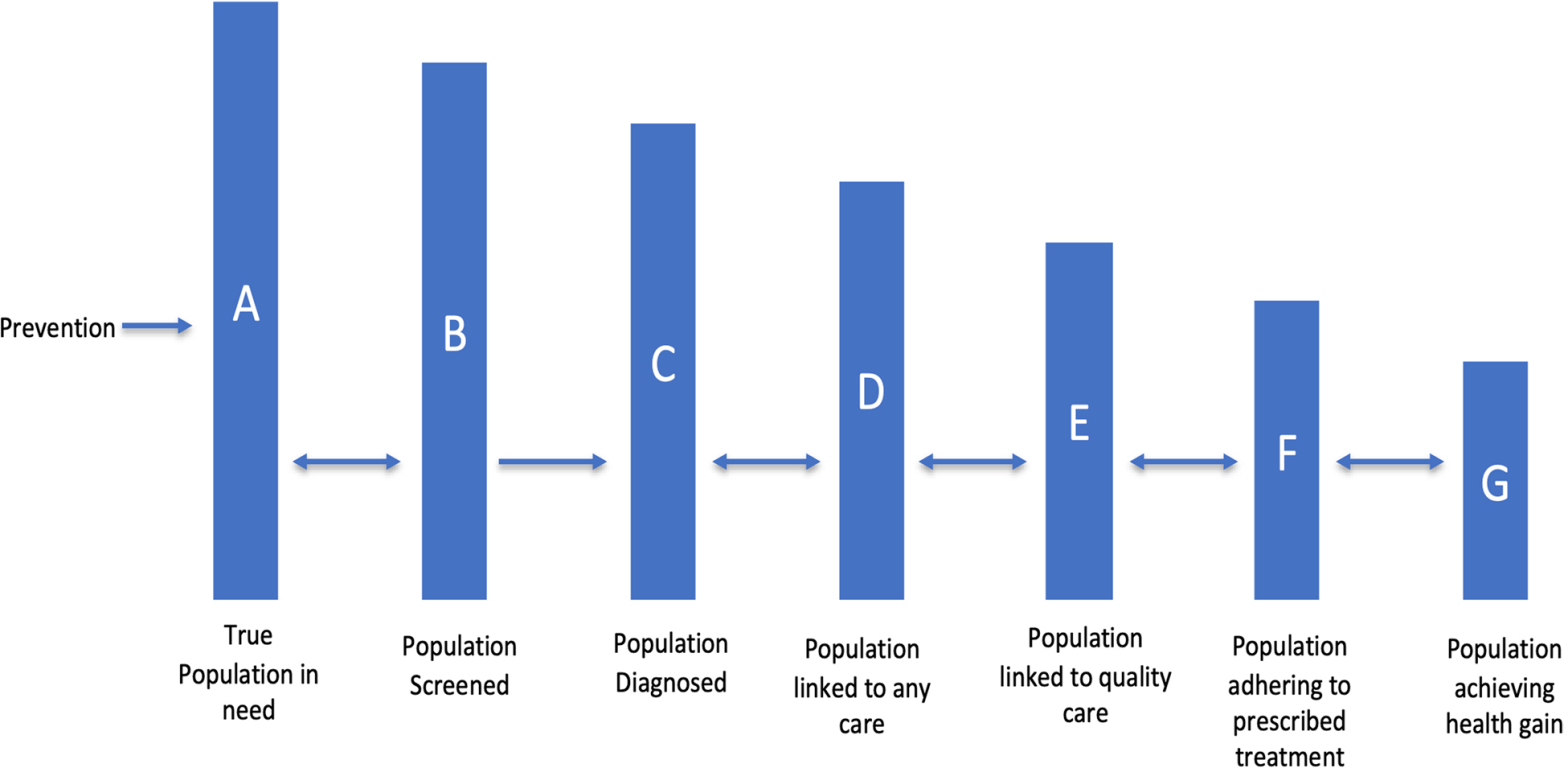
Proposed expanded hypertension care cascade

An expanded cascade of hypertension care is proposed that builds on previous standardized frameworks by incorporating additional steps to indicate the effectiveness of screening and treatment services provided by the health system (Fig. 3). Three new steps are proposed in the expanded hypertension care cascade: the percent of hypertensives that have ever had their blood pressure measured (to reflect facility readiness through frequency of blood pressure screening and early diagnosis), the percent of hypertensives linked to quality care (to reflect the content of care provided by the health system), and adherence to prescribed treatment regimens. From the expanded hypertension care cascade, an estimate of effective coverage (via quality-adjusted service coverage) can be calculated by taking the percent of individuals linked to quality care over the true population in need. Quality, use and need as applied to the expanded hypertension care cascade should be heuristically defined based on the health services delivery system in question, and the prioritized interventions for hypertension management being provided within that system.

While the proposed framework does not yet include standardized metrics for measuring quality of care or patient adherence, some potential measurement methods are proposed based on the results of this review (Table 3). Linkage to quality care may refer to the availability of drugs and blood pressure monitoring devices (structural quality), provider fidelity to standard treatment guidelines including prescribing practices and patient adherence (process quality), and/or patient satisfaction (outcome quality other than blood pressure control) among others. These can be measured by including additional questions in population-based surveys or through studies that link findings from household surveys (which provide information on service utilization and health outcomes) and facility-based surveys (which provide information on service quality). Patient adherence can include retention in care over time, adherence to lifestyle modification advice, and/or adherence to medication. These can be incorporated as additional questions in household surveys or through more complex methods like pill counts or treatment diaries. Even without standardized measurements of quality of care and patient adherence, it is hoped that the proposed framework can promote the consideration of intermediate outcomes such as fidelity to treatment protocols and regimens when examining population coverage of hypertension services. This consideration will also help to advance the conceptualization of process quality within effective coverage of hypertension management services, contributing to a standardized metric which will help improve health systems performance measurement.

**Table 3:** Proposed expanded hypertension care cascade description

Cascade Steps	Description	Proposed Measurement Techniques	Previous Studies that report this step in the care cascade	Notes and Considerations
True population in need (A)	Percent of population with blood pressure > 140/90 mmHg or previously correctly diagnosed as hypertensive	Cross-sectional and longitudinal population-based surveys with biometric measurements	Part of the existing care cascade	A high blood pressure reading at one point in time is not sufficient to diagnose hypertension. Cross-sectional studies that classify hypertensives based on one high blood pressure reading may be over-estimating the size of the population in need
Population screened (B)	Percent of population with high blood pressure who have had previously had blood pressure measured according to standards	Cross-sectional and longitudinal population-based surveys based on self-report. Linked patient observations/facility records to determine how often providers measure patient blood pressure	[22]	Population beyond those in need (A) should be screened for high blood pressure, however for the cascade framework, it is important to understand how many of those in need of services were previously screened. Individuals may also need to be screened more or less frequently based on other risk factors (e.g. age or comorbidities)
Population diagnosed (C)	Percent of population with high blood pressure who were previously diagnosed by a health worker	Cross-sectional and longitudinal population-based surveys based on self-report. Linked facility records to determine number of hypertensive patients	Part of the existing care cascade	Often referred to as the population “aware” of their condition. If providers are diagnosing non-hypertensive patients (false positives), the population diagnosed and true population in need (A and C) could be over-estimated
Population linked to any care (D)	Percent of population with high blood pressure who are linked to any treatment	Cross-sectional and longitudinal population-based surveys based on self-report	Part of the existing care cascade	Previously referred to as the population “treated” or receiving any treatment for hypertension. Discrepancies can arise from differences in definitions of contact coverage (e.g. taking any medication vs interactions with health providers)
Population receiving hypertension management services according to standards (E)	Percent of population with high blood pressure who are linked to quality treatment	Cross-sectional and longitudinal population-based surveys including the drugs prescribed. Linked facility records to determine quality of hypertension care provided	[38, 44]	This estimate requires some incorporation of a definition of “quality” of hypertension treatment. For standardization purposes, fidelity to national/global treatment guidelines would be the best way to assess service quality
Population adhering to treatment (F)	Percent of population with high blood pressure receiving quality treatment and adhering to treatment as prescribed	Cross-sectional and longitudinal population-based surveys potentially including pill counts or diaries	[41, 46]	Adherence to medications and/or lifestyle advice could be considered in this step
Population achieving health gain (G)	Percent of hypertensive population with controlled blood pressure	Cross-sectional and longitudinal population-based surveys with biometric measurements	Part of the existing care cascade	Health gain can be defined in multiple ways (e.g. controlled blood pressure levels, improved health, reduced hospitalization)

## Discussion

Hypertension is now more prevalent in LMICs than high-income countries, contributing to 7.5 million associated deaths in these countries each year [50]. Despite the massive burden of hypertension, only six studies have attempted to measure the effective coverage of hypertension management services in LMICs since 2000. This is a dearth of research relative to the 36 studies reporting effective coverage for reproductive, maternal, neonatal, and child health-related interventions found by a contemporary review [6]. The large difference in estimated crude and effective coverage within countries indicates the massive variability introduced when effective coverage for hypertension management services is calculated using non-standardized methods.

Researchers more frequently employ the hypertension care cascade than effective coverage to identify bottlenecks in achieving effective hypertension management; however, studies that provide insight into structural and process quality for hypertension management services received by populations are limited. In LMICs, the two largest gaps in the standard hypertension care are in the diagnosis of hypertension and achieving blood pressure control after treatment [20]. There is a critical need to scale up systemic strategies and interventions to target strategic points along the care cascade to improve population blood pressure control. Gaps remain in the standard hypertension care cascade, as the cascade measures the coverage of hypertension *control*, without accounting for the effect of health system-related *services* that contribute to effective management. This is the first study to systematically categorize the service quality-related aspects of the care cascade and propose an expanded care cascade based on these findings. Expanding the care cascade framework to incorporate measures of both screening- and treatment-related process quality will help directly identify service bottlenecks across the continuum of hypertension management services. This expanded framework also bridges the gap between the often-reported care cascade and the emerging conceptualization of effective coverage by providing an indication of quality-adjusted coverage of hypertension management services.

Previous efforts have characterized steps in care cascades where health benefits can be lost on the pathway to effective coverage; however, these have not been applied to hypertension care cascades [4, 6, 12]. The proposed new steps enable the quantification of missed opportunities for hypertension management based on access to care and the calculation of quality-adjusted coverage (E/A) and user-adherence-adjusted coverage of hypertension management services (F/A). They also enable a more comprehensive approach to studying effective coverage of these services beyond health outcomes alone.

There are some potential drawbacks to the expanded hypertension care cascade. One of the major bottlenecks described in the reviewed studies was a lack of facility readiness and structural quality. The percent of population ever screened (step B) is envisioned to be an indicator of facility readiness (to provide blood pressure screening services). However, there are shortcomings in this step’s ability to fully describe facility readiness. For example, the proposed step does not indicate how recently the individual has been screened for high blood pressure, which has implications for timely diagnosis of hypertension. Further, it does not indicate the quality of the screening services provided (e.g., whether correct cuff size is used, whether blood pressure measured twice) which has a large influence on whether or not a correct diagnosis is made. Another drawback is that certain steps are linked to locally relevant factors. Specifically, step E relies on hypertension management services being provided according to standards, which may vary locally, and step G may rely on a context-specific definition of non-elevated blood pressure (e.g., 140/90 mmHg vs 130/80 mmHg based on locally accepted guidelines). The proposed expanded care cascade should be further discussed by a global team of researchers to reach consensus on how to operationalize this framework in future research.

Evidence for the expanded hypertension care cascade came from studies conducted in 12 countries across five of the six WHO regions. To ensure consistency in future reporting of effective coverage of hypertension management, it will be important to incorporate perspectives of patients, researchers, and policymakers from multiple contexts when agreeing on international guidelines. As demonstrated by this review, there is currently no consensus among researchers on what constitutes effective coverage of hypertension management services. Further, the expanded hypertension care cascade can be applied to high-income countries, which similarly face challenges in providing effective hypertension management; fewer than half of hypertensive men and women achieve blood pressure control in high-income countries [51]. In the full-text review, two articles from Japan also reported gaps in the effective coverage of hypertension management services, emphasizing the need for additional research in high-income countries [52, 53]. Consensus across countries from multiple income groups and geographies will be necessary to track progress towards health systems functions that effectively manage a major contributor to the global burden of disease.

This study should be considered within its limitations. First, the search strategy excluded studies that did not provide population-representative estimates of hypertension management service coverage. Qualitative studies that examined the extent of provider knowledge relevant to hypertension treatment were therefore excluded [54, 55]. Several representative facility-based studies examined aspects of quality hypertension care, but without linking to a population-level survey, the percent of the population receiving these services, and thus the effective coverage, was unknown [56, 57]. Such linked study designs are common in the maternal and child health literature and should be increasingly used to determine quality-adjusted coverage for non-communicable disease management [11, 58–60]. Existing national household and facility-based surveys can be redesigned to encourage greater interoperability, which would add value to the hypertension care cascade and other program delivery analyses by enabling linked supply- and demand-side analysis. Second, the final results did not include studies published in languages other than English. At least one study was found in Spanish that included a definition of effective coverage of hypertension but was excluded [61]. Due to commonalities in authorship and study area with another included article, it is likely that the findings from this article are reflected in the results [27]. Third, this study is a review of the quality of hypertension management services, as characterized by the Donabedian quality framework, and is not intended to be a comprehensive review of all issues related to measuring effective coverage or quality. With new guidelines suggesting that the ideal threshold blood pressure is under 130/80 mmHg, the population in need of hypertension services and the hypertensive population with controlled blood pressure will drastically change [62]. Studies that have examined the effects of applying these guidelines report increases in hypertension prevalence ranging from 17.6% to 23.8% [63–65]. Additionally, certain aspects of quality such as equity, patient-centeredness, and efficiency are not comprehensively addressed in this review [66]. Future studies can apply the expanded cascade of care to different population sub-groups to enable equity analyses on receipt of quality services, and link cascades with information on health system expenditure and patient perspectives on care to reveal the effects of various guidelines and reforms on effective coverage.

## Conclusion

This study reviewed the evidence on effective coverage for hypertension management and more broadly, quality within the hypertension care cascade. Although there is no consensus definition of effective coverage and indicators of quality vary by study, there are some common approaches to describing barriers on the pathway to effective coverage of hypertension management services. These approaches have been incorporated into an expanded hypertension care cascade framework that considers aspects of structural and process quality. Future studies should incorporate aspects of service quality in population measures of hypertension management coverage in LMICs. It is also necessary to improve our understanding of how interventions can improve intermediate outcomes in hypertension management (e.g. expansion of screening services, fidelity to treatment guidelines, and medication adherence). These studies are essential for understanding how to best align interventions and health systems to combat the high prevalence of hypertension in LMICs. This approach of studying effective coverage and quality-adjusted cascades of care helps to advance measurement of health systems performance, ultimately improving the quality of life for people with chronic diseases living in LMICs.

## Supplementary Information

**Additional file 1:**  Approaches for measuring effective coverage

**Additional file 2: **Scoping review search strategy

**Additional file 3:  **Quality assessment for studies included in framework development 

**Additional file 4:**  World bank reports that incorporate measures of service quality to measures of hypertension service coverage 

**Additional file 5: **Full-text review dataset description of data

## Data Availability

The citation information of all articles included in the full-text review is included in Additional file 5. Additional materials, such as data collection forms, data extracted from included studies, data used for all analyses, and analytic code are available upon reasonable request to the corresponding author.

## Abbreviations

AXIS: Appraisal tool for Cross-Sectional Studies
DTP: Diphtheria, Tetanus, and Pertussis
HTN: Hypertension
LMICs: Low- and Middle-Income Countries
NCDs: Non-Communicable Diseases
PRISMA: Preferred Reporting Items for Systematic Reviews

## Footnotes

1 Specifically, hypertension is defined as an individual with systolic blood pressure of 140 mm Hg or more and/or diastolic blood pressure of 90 mm Hg or more and/or taking antihypertensive medication.
