# Supplementary material for: Evidence for an expanded hypertension care cascade in low- and middle-income countries: a scoping review
Source: BMC Health Serv Res. 2022 Jun 27;22:827. doi: 10.1186/s12913-022-08190-0 (PMC9235242; doi:10.1186/s12913-022-08190-0)
Supplement: Supplementary file 1 — Additional file 1: Approaches for measuring effective coverage [file 12913_2022_8190_MOESM1_ESM.pdf]

Annex 1: Approaches for Measuring Effective Coverage

Approach	Description	Study examples	Potential data sources	Strengths	Limitations
Content of care	Focuses on the health care process<br><br>Involves indicators that target the resource and activity outputs of an intervention	Effective coverage of primary health care services in 8 countries<br><br>(Leslie *et al.*, 2017)	Direct observation or clinical vignettes<br><br>Hospital databases<br><br>Patient exit interviews	Offers information from both demand- and supply-side factors<br><br>Resource and activity outputs can serve as objective indicators	Subjectivity in patient assessments of quality<br><br>High outputs or content of care may not directly translate into health gains
Biomarkers	Focuses on the health benefits that can be detected biologically	Assessment of vaccine effectiveness<br><br>(Pebody *et al.*, 2002)	Health surveys that include physical examinations	Provides an objective measure of actual health gains or impact	Collection of biomarker data can be costly and not always feasible in resource-constrained settings<br><br>Not applicable to all health conditions
Cohort registration	Focuses on changes in individual health outcomes over the course of treatment	Assessment of highly active antiretroviral therapy (HAART)<br><br>(Sterne *et al.*, 2005)	Cohort registration databases	Provides measurement of treatment effectiveness for chronic conditions over time	Limited to interventions that involve close patient monitoring and treatment by healthcare providers<br><br>Requires careful consideration of time-dependent confounding factors and lost to follow-up
Exposure matching	Compares health outcomes of individuals who had intervention exposure to those who did not have exposure to an intervention	Assessment of health impact of IPTp and ITNs<br><br>(Eisele *et al.*, 2012)	Household survey data	Allows for the quantification of the health gains associated with intervention exposure by calculating odds ratios or relative risks with existing data	Household surveys are rarely powered to detect health effects Unmeasured confounding factors need to be accounted for due to the observational nature of analysis
Statistical methods	Uses statistical and econometric techniques, such as instrumental variables and matching, to estimate health outcomes while controlling for unobserved variables	Assessment of diabetes and hypertension management in Iran<br><br>(Farzadfar *et al.*, 2012)	Health survey data	Offers a convenient solution to address potential biases associated with confounding factors	Only approximates the relationship, or correlation, between intervention exposure and a health outcome rather than the causal effect
Risk-adjusted outcomes	Estimates health outcomes while accounting for the patient characteristics and risks of death that can vary systematically across sites	Birth weight–adjusted neonatal mortality<br><br>(Straney, Lim and Murray, 2012)	Hospital databases	Provides an indicator for quality of care that reflects both procedural outputs and the health impact of received care	Limited to interventions that are delivered at health facilities- Certain risks may not be easily adjusted for if they are challenging to quantify

Adapted from (Ng *et al.*, 2014) *and* (Jannati *et al.*, 2018)
