# Supplementary material for: Evidence for an expanded hypertension care cascade in low- and middle-income countries: a scoping review
Source: BMC Health Serv Res. 2022 Jun 27;22:827. doi: 10.1186/s12913-022-08190-0 (PMC9235242; doi:10.1186/s12913-022-08190-0)
Supplement: Supplementary file 2 — Additional file 2: Scoping review search strategy [file 12913_2022_8190_MOESM2_ESM.pdf]

Annex 2: Scoping Review Search Strategy

Search conducted on 12 October, 2020

Database	Effective Coverage	N	Care Cascade	N	Combined	N
SCOPUS	TITLE-ABS-KEY (“effective coverage” AND “hypertension”)	13	TITLE-ABS-KEY (“effective coverage” AND “hypertension”)	201	TITLE-ABS-KEY (hypertension) AND (TITLE-ABS-KEY ("effective coverage") OR (TITLE-ABS-KEY (cascade AND care)))	218
EMBASE	**hypertension**:ti,ab,kw AND **‘effective coverage’**:ti,ab,kw	15	**hypertension**:ti,ab,kw AND **cascade**:ti,ab,kw AND **care**:ti,ab,kw	121	hypertension:ti,ab,kw AND 'effective coverage':ti,ab,kw OR 'care cascade':ti,ab,kw OR 'cascade of care':ti,ab,kw	892
PubMed	(hypertension) AND (‘effective coverage’)	492	(hypertension) AND (care) AND (cascade)	158	(hypertension) AND ((care) AND (cascade)) OR ("effective coverage")	486
ScienceDirect	“hypertension” AND “effective coverage”	81	Title, abstract, keywords: “hypertension” AND “cascade”	363	“hypertension” AND “effective coverage” OR ("care cascade" OR "cascade of care")	719
ProQuest	hypertension AND “effective coverage”	370	noft(hypertension) AND care AND noft(cascade)	397	noft(hypertension) AND noft("effective coverage") OR noft("care cascade" OR "cascade of care")	510
Web of Science	**ALL FIELDS:** (hypertension) *AND* **ALL FIELDS:** ("effective coverage")	13	**ALL FIELDS:** (hypertension) *AND* **ALL FIELDS:** ("care cascade") *OR* **ALL FIELDS:** ("cascade of care")	289	**ALL FIELDS:** (hypertension) *AND* **ALL FIELDS:** ("care cascade") *OR* **ALL FIELDS:** ("cascade of care") *OR* **ALL FIELDS:** ("effective coverage")	881

Search conducted on 26 October, 2020 to add hypertension prevalence, awareness, control to search

https://epoc.cochrane.org/lmic-filters is the source of tags for LMICs

Searched for “hypertension” AND “prevalence” OR “awareness” AND “treatment” and “control” and “LMIC” in title/abstract

Embase search terms below- 589 articles

hypertension:ab,ti AND (prevalence:ab,ti OR awareness:ab,ti) AND treatment:ab,ti AND control:ab,ti AND (afghanistan:ti,ab,kw OR albania:ti,ab,kw OR algeria:ti,ab,kw OR (american:ti,ab,kw AND samoa:ti,ab,kw) OR angola:ti,ab,kw OR 'antigua and barbuda':ti,ab,kw OR antigua:ti,ab,kw OR barbuda:ti,ab,kw OR argentina:ti,ab,kw OR armenia:ti,ab,kw OR armenian:ti,ab,kw OR aruba:ti,ab,kw OR azerbaijan:ti,ab,kw OR bahrain:ti,ab,kw OR bangladesh:ti,ab,kw OR barbados:ti,ab,kw OR (republic:ti,ab,kw AND of:ti,ab,kw AND belarus:ti,ab,kw) OR belarus:ti,ab,kw OR byelarus:ti,ab,kw OR belorussia:ti,ab,kw OR byelorussian:ti,ab,kw OR belize:ti,ab,kw OR (british:ti,ab,kw AND

honduras:ti,ab,kw) OR benin:ti,ab,kw OR dahomey:ti,ab,kw OR bhutan:ti,ab,kw OR bolivia:ti,ab,kw OR 'bosnia and herzegovina':ti,ab,kw OR bosnia:ti,ab,kw OR herzegovina:ti,ab,kw OR botswana:ti,ab,kw OR bechuanaland:ti,ab,kw OR brazil:ti,ab,kw OR brasil:ti,ab,kw OR bulgaria:ti,ab,kw OR (burkina:ti,ab,kw AND faso:ti,ab,kw) OR (burkina:ti,ab,kw AND fasso:ti,ab,kw) OR (upper:ti,ab,kw AND volta:ti,ab,kw) OR burundi:ti,ab,kw OR urundi:ti,ab,kw OR (cabo:ti,ab,kw AND verde:ti,ab,kw) OR (cape:ti,ab,kw AND verde:ti,ab,kw) OR cambodia:ti,ab,kw OR kampuchea:ti,ab,kw OR (khmer:ti,ab,kw AND republic:ti,ab,kw) OR cameroon:ti,ab,kw OR cameron:ti,ab,kw OR cameroun:ti,ab,kw OR (central:ti,ab,kw AND african:ti,ab,kw AND republic:ti,ab,kw) OR (ubangi:ti,ab,kw AND shari:ti,ab,kw) OR chad:ti,ab,kw OR chile:ti,ab,kw OR china:ti,ab,kw OR colombia:ti,ab,kw OR comoros:ti,ab,kw OR (comoro:ti,ab,kw AND islands:ti,ab,kw) OR (iles:ti,ab,kw AND comores:ti,ab,kw) OR mayotte:ti,ab,kw OR (democratic:ti,ab,kw AND republic:ti,ab,kw AND of:ti,ab,kw AND the:ti,ab,kw AND congo:ti,ab,kw) OR (democratic:ti,ab,kw AND republic:ti,ab,kw AND congo:ti,ab,kw) OR congo:ti,ab,kw OR zaire:ti,ab,kw OR (costa:ti,ab,kw AND rica:ti,ab,kw) OR (`cote:ti,ab,kw AND d:ti,ab,kw AND 'ivoire':ti,ab,kw) OR (`cote:ti,ab,kw AND d:ti,ab,kw AND 'ivoire':ti,ab,kw) OR (cote:ti,ab,kw AND divoire:ti,ab,kw) OR (cote:ti,ab,kw AND d:ti,ab,kw AND ivoire:ti,ab,kw) OR (ivory:ti,ab,kw AND coast:ti,ab,kw) OR croatia:ti,ab,kw OR cuba:ti,ab,kw OR cyprus:ti,ab,kw OR (czech:ti,ab,kw AND republic:ti,ab,kw) OR czechoslovakia:ti,ab,kw OR djibouti:ti,ab,kw OR (french:ti,ab,kw AND somaliland:ti,ab,kw) OR dominica:ti,ab,kw OR (dominican:ti,ab,kw AND republic:ti,ab,kw) OR ecuador:ti,ab,kw OR egypt:ti,ab,kw OR (united:ti,ab,kw AND arab:ti,ab,kw AND republic:ti,ab,kw) OR (el:ti,ab,kw AND salvador:ti,ab,kw) OR (equatorial:ti,ab,kw AND guinea:ti,ab,kw) OR (spanish:ti,ab,kw AND guinea:ti,ab,kw) OR eritrea:ti,ab,kw OR estonia:ti,ab,kw OR eswatini:ti,ab,kw OR swaziland:ti,ab,kw OR ethiopia:ti,ab,kw OR fiji:ti,ab,kw OR gabon:ti,ab,kw OR (gabonese:ti,ab,kw AND republic:ti,ab,kw) OR gambia:ti,ab,kw OR 'georgia (republic)':ti,ab,kw OR georgian:ti,ab,kw OR ghana:ti,ab,kw OR (gold:ti,ab,kw AND coast:ti,ab,kw) OR gibraltar:ti,ab,kw OR greece:ti,ab,kw OR grenada:ti,ab,kw OR guam:ti,ab,kw OR guatemala:ti,ab,kw OR (guinea:ti,ab,kw AND bissau:ti,ab,kw) OR guyana:ti,ab,kw OR (british:ti,ab,kw AND guiana:ti,ab,kw) OR haiti:ti,ab,kw OR hispaniola:ti,ab,kw OR honduras:ti,ab,kw OR hungary:ti,ab,kw OR india:ti,ab,kw OR indonesia:ti,ab,kw OR timor:ti,ab,kw OR iran:ti,ab,kw OR iraq:ti,ab,kw OR (isle:ti,ab,kw AND of:ti,ab,kw AND man:ti,ab,kw) OR jamaica:ti,ab,kw OR jordan:ti,ab,kw OR kazakhstan:ti,ab,kw OR kazakh:ti,ab,kw OR kenya:ti,ab,kw OR (`democratic:ti,ab,kw AND people:ti,ab,kw AND 's republic of korea':ti,ab,kw) OR (republic:ti,ab,kw AND of:ti,ab,kw AND korea:ti,ab,kw) OR (north:ti,ab,kw AND korea:ti,ab,kw) OR (south:ti,ab,kw AND korea:ti,ab,kw) OR korea:ti,ab,kw OR kosovo:ti,ab,kw OR kyrgyzstan:ti,ab,kw OR kirghizia:ti,ab,kw OR kirgizstan:ti,ab,kw OR (kyrgyz:ti,ab,kw AND republic:ti,ab,kw) OR kirghiz:ti,ab,kw OR laos:ti,ab,kw OR (lao:ti,ab,kw AND pdr:ti,ab,kw) OR (`lao:ti,ab,kw AND people:ti,ab,kw AND 's democratic republic':ti,ab,kw) OR latvia:ti,ab,kw OR lebanon:ti,ab,kw OR (lebanese:ti,ab,kw AND republic:ti,ab,kw) OR lesotho:ti,ab,kw OR basutoland:ti,ab,kw OR liberia:ti,ab,kw OR libya:ti,ab,kw OR (libyan:ti,ab,kw AND arab:ti,ab,kw AND jamahiriya:ti,ab,kw) OR lithuania:ti,ab,kw OR macau:ti,ab,kw OR macao:ti,ab,kw OR (republic:ti,ab,kw AND of:ti,ab,kw AND north:ti,ab,kw AND macedonia:ti,ab,kw) OR macedonia:ti,ab,kw OR madagascar:ti,ab,kw OR (malagasy:ti,ab,kw AND republic:ti,ab,kw) OR malawi:ti,ab,kw OR nyasaland:ti,ab,kw OR malaysia:ti,ab,kw OR (malay:ti,ab,kw AND federation:ti,ab,kw) OR (malaya:ti,ab,kw AND federation:ti,ab,kw) OR maldives:ti,ab,kw OR (indian:ti,ab,kw AND ocean:ti,ab,kw AND islands:ti,ab,kw) OR (indian:ti,ab,kw AND ocean:ti,ab,kw) OR mali:ti,ab,kw OR malta:ti,ab,kw OR micronesia:ti,ab,kw OR (federated:ti,ab,kw AND states:ti,ab,kw AND of:ti,ab,kw AND micronesia:ti,ab,kw) OR kiribati:ti,ab,kw OR (marshall:ti,ab,kw AND islands:ti,ab,kw) OR nauru:ti,ab,kw OR (northern:ti,ab,kw AND mariana:ti,ab,kw AND islands:ti,ab,kw) OR palau:ti,ab,kw OR tuvalu:ti,ab,kw OR mauritania:ti,ab,kw OR mauritius:ti,ab,kw OR mexico:ti,ab,kw OR moldova:ti,ab,kw OR moldovian:ti,ab,kw OR mongolia:ti,ab,kw OR montenegro:ti,ab,kw OR 'montenegro (republic)':ti,ab,kw OR morocco:ti,ab,kw OR ifni:ti,ab,kw OR mozambique:ti,ab,kw OR (portuguese:ti,ab,kw AND east:ti,ab,kw AND africa:ti,ab,kw) OR myanmar:ti,ab,kw OR burma:ti,ab,kw OR namibia:ti,ab,kw OR nepal:ti,ab,kw OR (netherlands:ti,ab,kw AND antilles:ti,ab,kw) OR nicaragua:ti,ab,kw OR niger:ti,ab,kw OR nigeria:ti,ab,kw OR oman:ti,ab,kw OR muscat:ti,ab,kw OR

pakistan:ti,ab,kw OR panama:ti,ab,kw OR (papua:ti,ab,kw AND new:ti,ab,kw AND guinea:ti,ab,kw) OR (new:ti,ab,kw AND guinea:ti,ab,kw) OR paraguay:ti,ab,kw OR peru:ti,ab,kw OR philippines:ti,ab,kw OR philipines:ti,ab,kw OR phillipines:ti,ab,kw OR phillippines:ti,ab,kw OR poland:ti,ab,kw OR (`polish:ti,ab,kw AND people:ti,ab,kw AND 's republic':ti,ab,kw) OR portugal:ti,ab,kw OR (portuguese:ti,ab,kw AND republic:ti,ab,kw) OR (puerto:ti,ab,kw AND rico:ti,ab,kw) OR romania:ti,ab,kw OR russia:ti,ab,kw OR (russian:ti,ab,kw AND federation:ti,ab,kw) OR ussr:ti,ab,kw OR (soviet:ti,ab,kw AND union:ti,ab,kw) OR (union:ti,ab,kw AND of:ti,ab,kw AND soviet:ti,ab,kw AND socialist:ti,ab,kw AND republics:ti,ab,kw) OR rwanda:ti,ab,kw OR ruanda:ti,ab,kw OR samoa:ti,ab,kw OR (pacific:ti,ab,kw AND islands:ti,ab,kw) OR polynesia:ti,ab,kw OR (samoan:ti,ab,kw AND islands:ti,ab,kw) OR (navigator:ti,ab,kw AND island:ti,ab,kw) OR (navigator:ti,ab,kw AND islands:ti,ab,kw) OR 'sao tome and principe':ti,ab,kw OR (saudi:ti,ab,kw AND arabia:ti,ab,kw) OR senegal:ti,ab,kw OR serbia:ti,ab,kw OR seychelles:ti,ab,kw OR (sierra:ti,ab,kw AND leone:ti,ab,kw) OR slovakia:ti,ab,kw OR (slovak:ti,ab,kw AND republic:ti,ab,kw) OR slovenia:ti,ab,kw OR melanesia:ti,ab,kw OR (solomon:ti,ab,kw AND island:ti,ab,kw) OR (solomon:ti,ab,kw AND islands:ti,ab,kw) OR (norfolk:ti,ab,kw AND island:ti,ab,kw) OR (norfolk:ti,ab,kw AND islands:ti,ab,kw) OR somalia:ti,ab,kw OR (south:ti,ab,kw AND africa:ti,ab,kw) OR (south:ti,ab,kw AND sudan:ti,ab,kw) OR (sri:ti,ab,kw AND lanka:ti,ab,kw) OR ceylon:ti,ab,kw OR 'saint kitts and nevis':ti,ab,kw OR 'st. kitts and nevis':ti,ab,kw OR (saint:ti,ab,kw AND lucia:ti,ab,kw) OR 'st. lucia':ti,ab,kw OR 'saint vincent and the grenadines':ti,ab,kw OR (saint:ti,ab,kw AND vincent:ti,ab,kw) OR 'st. vincent':ti,ab,kw OR grenadines:ti,ab,kw OR sudan:ti,ab,kw OR suriname:ti,ab,kw OR surinam:ti,ab,kw OR (dutch:ti,ab,kw AND guiana:ti,ab,kw) OR (netherlands:ti,ab,kw AND guiana:ti,ab,kw) OR syria:ti,ab,kw OR (syrian:ti,ab,kw AND arab:ti,ab,kw AND republic:ti,ab,kw) OR tajikistan:ti,ab,kw OR tadjikistan:ti,ab,kw OR tadzhikistan:ti,ab,kw OR tadzhik:ti,ab,kw OR tanzania:ti,ab,kw OR tanganyika:ti,ab,kw OR thailand:ti,ab,kw OR siam:ti,ab,kw OR (timor:ti,ab,kw AND leste:ti,ab,kw) OR (east:ti,ab,kw AND timor:ti,ab,kw) OR togo:ti,ab,kw OR (togolese:ti,ab,kw AND republic:ti,ab,kw) OR tonga:ti,ab,kw OR 'trinidad and tobago':ti,ab,kw OR trinidad:ti,ab,kw OR tobago:ti,ab,kw OR tunisia:ti,ab,kw OR 'turkey (republic)':ti,ab,kw OR turkey:ti,ab,kw OR turkmenistan:ti,ab,kw OR turkmen:ti,ab,kw OR uganda:ti,ab,kw OR ukraine:ti,ab,kw OR uruguay:ti,ab,kw OR uzbekistan:ti,ab,kw OR uzbek:ti,ab,kw OR vanuatu:ti,ab,kw OR (new:ti,ab,kw AND hebrides:ti,ab,kw) OR venezuela:ti,ab,kw OR vietnam:ti,ab,kw OR (viet:ti,ab,kw AND nam:ti,ab,kw) OR (middle:ti,ab,kw AND east:ti,ab,kw) OR (west:ti,ab,kw AND bank:ti,ab,kw) OR gaza:ti,ab,kw OR palestine:ti,ab,kw OR yemen:ti,ab,kw OR yugoslavia:ti,ab,kw OR zambia:ti,ab,kw OR zimbabwe:ti,ab,kw OR (northern:ti,ab,kw AND rhodesia:ti,ab,kw) OR (global:ti,ab,kw AND south:ti,ab,kw) OR (africa:ti,ab,kw AND south:ti,ab,kw AND of:ti,ab,kw AND the:ti,ab,kw AND sahara:ti,ab,kw) OR 'sub saharan africa':ti,ab,kw OR (subsaharan:ti,ab,kw AND africa:ti,ab,kw) OR (africa,:ti,ab,kw AND central:ti,ab,kw) OR (central:ti,ab,kw AND africa:ti,ab,kw) OR (africa,:ti,ab,kw AND northern:ti,ab,kw) OR (north:ti,ab,kw AND africa:ti,ab,kw) OR (northern:ti,ab,kw AND africa:ti,ab,kw) OR magreb:ti,ab,kw OR maghrib:ti,ab,kw OR sahara:ti,ab,kw OR (africa,:ti,ab,kw AND southern:ti,ab,kw OR southern:ti,ab,kw) AND africa:ti,ab,kw OR (africa,:ti,ab,kw AND eastern:ti,ab,kw) OR (east:ti,ab,kw AND africa:ti,ab,kw) OR (eastern:ti,ab,kw AND africa:ti,ab,kw) OR (africa,:ti,ab,kw AND western:ti,ab,kw) OR (west:ti,ab,kw AND africa:ti,ab,kw) OR (western:ti,ab,kw AND africa:ti,ab,kw) OR (west:ti,ab,kw AND indies:ti,ab,kw) OR (indian:ti,ab,kw AND ocean:ti,ab,kw AND islands:ti,ab,kw) OR (caribbean:ti,ab,kw AND region:ti,ab,kw) OR (caribbean:ti,ab,kw AND islands:ti,ab,kw) OR caribbean:ti,ab,kw OR (central:ti,ab,kw AND america:ti,ab,kw) OR (latin:ti,ab,kw AND america:ti,ab,kw) OR 'south and central america':ti,ab,kw OR (south:ti,ab,kw AND america:ti,ab,kw) OR (asia,:ti,ab,kw AND central:ti,ab,kw) OR (central:ti,ab,kw AND asia:ti,ab,kw) OR (asia,:ti,ab,kw AND northern:ti,ab,kw) OR (north:ti,ab,kw AND asia:ti,ab,kw) OR (northern:ti,ab,kw AND asia:ti,ab,kw) OR (asia,:ti,ab,kw AND southeastern:ti,ab,kw) OR (southeastern:ti,ab,kw AND asia:ti,ab,kw) OR (south:ti,ab,kw AND eastern:ti,ab,kw AND asia:ti,ab,kw) OR (southeast:ti,ab,kw AND asia:ti,ab,kw) OR (south:ti,ab,kw AND east:ti,ab,kw AND asia:ti,ab,kw) OR (asia,:ti,ab,kw AND western:ti,ab,kw) OR (western:ti,ab,kw AND asia:ti,ab,kw) OR (europe,:ti,ab,kw AND eastern:ti,ab,kw) OR (east:ti,ab,kw

AND europe:ti,ab,kw) OR (eastern:ti,ab,kw AND europe:ti,ab,kw) OR (developing:ti,ab,kw AND country:ti,ab,kw) OR (developing:ti,ab,kw AND countries:ti,ab,kw) OR (developing:ti,ab,kw AND nation?:ti,ab,kw) OR (developing:ti,ab,kw AND population?:ti,ab,kw) OR (developing:ti,ab,kw AND world:ti,ab,kw) OR (less:ti,ab,kw AND developed:ti,ab,kw AND countr*:ti,ab,kw) OR (less:ti,ab,kw AND developed:ti,ab,kw AND nation?:ti,ab,kw) OR (less:ti,ab,kw AND developed:ti,ab,kw AND population?:ti,ab,kw) OR (less:ti,ab,kw AND developed:ti,ab,kw AND world:ti,ab,kw) OR (lesser:ti,ab,kw AND developed:ti,ab,kw AND countr*:ti,ab,kw) OR (lesser:ti,ab,kw AND developed:ti,ab,kw AND nation?:ti,ab,kw) OR (lesser:ti,ab,kw AND developed:ti,ab,kw AND population?:ti,ab,kw) OR (lesser:ti,ab,kw AND developed:ti,ab,kw AND world:ti,ab,kw) OR (under:ti,ab,kw AND developed:ti,ab,kw AND countr*:ti,ab,kw) OR (under:ti,ab,kw AND developed:ti,ab,kw AND nation?:ti,ab,kw) OR (under:ti,ab,kw AND developed:ti,ab,kw AND population?:ti,ab,kw) OR (under:ti,ab,kw AND developed:ti,ab,kw AND world:ti,ab,kw) OR (underdeveloped:ti,ab,kw AND countr*:ti,ab,kw) OR (underdeveloped:ti,ab,kw AND nation?:ti,ab,kw) OR (underdeveloped:ti,ab,kw AND population?:ti,ab,kw) OR (underdeveloped:ti,ab,kw AND world:ti,ab,kw) OR (middle:ti,ab,kw AND income:ti,ab,kw AND countr*:ti,ab,kw) OR (middle:ti,ab,kw AND income:ti,ab,kw AND nation?:ti,ab,kw) OR (middle:ti,ab,kw AND income:ti,ab,kw AND population?:ti,ab,kw) OR (low:ti,ab,kw AND income:ti,ab,kw AND countr*:ti,ab,kw) OR (low:ti,ab,kw AND income:ti,ab,kw AND nation?:ti,ab,kw) OR (low:ti,ab,kw AND income:ti,ab,kw AND population?:ti,ab,kw) OR (lower:ti,ab,kw AND income:ti,ab,kw AND countr*:ti,ab,kw) OR (lower:ti,ab,kw AND income:ti,ab,kw AND nation?:ti,ab,kw) OR (lower:ti,ab,kw AND income:ti,ab,kw AND population?:ti,ab,kw) OR (underserved:ti,ab,kw AND countr*:ti,ab,kw) OR (underserved:ti,ab,kw AND nation?:ti,ab,kw) OR (underserved:ti,ab,kw AND population?:ti,ab,kw) OR (underserved:ti,ab,kw AND world:ti,ab,kw) OR (under:ti,ab,kw AND served:ti,ab,kw AND countr*:ti,ab,kw) OR (under:ti,ab,kw AND served:ti,ab,kw AND nation?:ti,ab,kw) OR (under:ti,ab,kw AND served:ti,ab,kw AND population?:ti,ab,kw) OR (under:ti,ab,kw AND served:ti,ab,kw AND world:ti,ab,kw) OR (deprived:ti,ab,kw AND countr*:ti,ab,kw) OR (deprived:ti,ab,kw AND nation?:ti,ab,kw) OR (deprived:ti,ab,kw AND population?:ti,ab,kw) OR (deprived:ti,ab,kw AND world:ti,ab,kw) OR (poor:ti,ab,kw AND countr*:ti,ab,kw) OR (poor:ti,ab,kw AND nation?:ti,ab,kw) OR (poor:ti,ab,kw AND population?:ti,ab,kw) OR (poor:ti,ab,kw AND world:ti,ab,kw) OR (poorer:ti,ab,kw AND countr*:ti,ab,kw) OR (poorer:ti,ab,kw AND nation?:ti,ab,kw) OR (poorer:ti,ab,kw AND population?:ti,ab,kw) OR (poorer:ti,ab,kw AND world:ti,ab,kw) OR (developing:ti,ab,kw AND econom*:ti,ab,kw) OR (less:ti,ab,kw AND developed:ti,ab,kw AND econom*:ti,ab,kw) OR (lesser:ti,ab,kw AND developed:ti,ab,kw AND econom*:ti,ab,kw) OR (under:ti,ab,kw AND developed:ti,ab,kw AND econom*:ti,ab,kw) OR (underdeveloped:ti,ab,kw AND econom*:ti,ab,kw) OR (middle:ti,ab,kw AND income:ti,ab,kw AND econom*:ti,ab,kw) OR (low:ti,ab,kw AND income:ti,ab,kw AND econom*:ti,ab,kw) OR (lower:ti,ab,kw AND income:ti,ab,kw AND econom*:ti,ab,kw) OR (low:ti,ab,kw AND gdp:ti,ab,kw) OR (low:ti,ab,kw AND gnp:ti,ab,kw) OR (low:ti,ab,kw AND gross:ti,ab,kw AND domestic:ti,ab,kw) OR (low:ti,ab,kw AND gross:ti,ab,kw AND national:ti,ab,kw) OR (lower:ti,ab,kw AND gdp:ti,ab,kw) OR (lower:ti,ab,kw AND gnp:ti,ab,kw) OR (lower:ti,ab,kw AND gross:ti,ab,kw AND domestic:ti,ab,kw) OR (lower:ti,ab,kw AND gross:ti,ab,kw AND national:ti,ab,kw) OR lmic:ti,ab,kw OR lmics:ti,ab,kw OR (third:ti,ab,kw AND world:ti,ab,kw) OR (lami:ti,ab,kw AND countr*:ti,ab,kw) OR (transitional:ti,ab,kw AND countr*:ti,ab,kw) OR (emerging:ti,ab,kw AND economies:ti,ab,kw) OR (emerging:ti,ab,kw AND nation?:ti,ab,kw))

Pubmed search terms below- 750 articles

(hypertension[Title/Abstract]) AND (prevalence[Title/Abstract] OR awareness[Title/Abstract]) AND (treatment[Title/Abstract]) AND (control[Title/Abstract]) AND (afghanistan[MeSH] OR albania[MeSH] OR algeria[MeSH] OR american samoa[MeSH] OR angola[MeSH] OR antigua and barbuda[MeSH] OR argentina[MeSH] OR armenia[MeSH] OR aruba[MeSH] OR azerbaijan[MeSH] OR bahrain[MeSH] OR

bangladesh[MeSH] OR barbados[MeSH] OR republic of belarus[MeSH] OR belize[MeSH] OR benin[MeSH] OR bhutan[MeSH] OR bolivia[MeSH] OR bosnia and herzegovina[MeSH] OR botswana[MeSH] OR brazil[MeSH] OR bulgaria[MeSH] OR burkina faso[MeSH] OR burundi[MeSH] OR cabo verde[MeSH] OR cambodia[MeSH] OR cameroon[MeSH] OR central african republic[MeSH] OR chad[MeSH] OR chile[MeSH] OR china[MeSH] OR colombia[MeSH] OR comoros[MeSH] OR democratic republic of the congo[MeSH] OR congo[MeSH] OR costa rica[MeSH] OR cote d'ivoire[MeSH] OR croatia[MeSH] OR cuba[MeSH] OR cyprus[MeSH] OR czech republic[MeSH] OR djibouti[MeSH] OR dominica[MeSH] OR dominican republic[MeSH] OR ecuador[MeSH] OR egypt[MeSH] OR el salvador[MeSH] OR equatorial guinea[MeSH] OR eritrea[MeSH] OR estonia[MeSH] OR swaziland[MeSH] OR ethiopia[MeSH] OR fiji[MeSH] OR gabon[MeSH] OR gambia[MeSH] OR georgia (republic)[MeSH] OR ghana[MeSH] OR gibraltar[MeSH] OR greece[MeSH] OR grenada[MeSH] OR guam[MeSH] OR guatemala[MeSH] OR guinea[MeSH] OR guinea bissau[MeSH] OR guyana[MeSH] OR haiti[MeSH] OR honduras[MeSH] OR hungary[MeSH] OR india[MeSH] OR indonesia[MeSH] OR iran[MeSH] OR iraq[MeSH] OR jamaica[MeSH] OR jordan[MeSH] OR kazakhstan[MeSH] OR kenya[MeSH] OR democratic people's republic of korea[MeSH] OR republic of korea[MeSH] OR kosovo[MeSH] OR kyrgyzstan[MeSH] OR laos[MeSH] OR latvia[MeSH] OR lebanon[MeSH] OR lesotho[MeSH] OR liberia[MeSH] OR libya[MeSH] OR lithuania[MeSH] OR macau[MeSH] OR republic of north macedonia[MeSH] OR madagascar[MeSH] OR malawi[MeSH] OR malaysia[MeSH] OR indian ocean islands[MeSH] OR mali[MeSH] OR malta[MeSH] OR micronesia[MeSH] OR palau[MeSH] OR mauritania[MeSH] OR mauritius[MeSH] OR mexico[MeSH] OR moldova[MeSH] OR mongolia[MeSH] OR montenegro[MeSH] OR morocco[MeSH] OR mozambique[MeSH] OR myanmar[MeSH] OR namibia[MeSH] OR nepal[MeSH] OR netherlands antilles[MeSH] OR nicaragua[MeSH] OR niger[MeSH] OR nigeria[MeSH] OR oman[MeSH] OR pakistan[MeSH] OR panama[MeSH] OR papua new guinea[MeSH] OR paraguay[MeSH] OR peru[MeSH] OR philippines[MeSH] OR poland[MeSH] OR portugal[MeSH] OR puerto rico[MeSH] OR romania[MeSH] OR russia[MeSH] OR rwanda[MeSH] OR samoa[MeSH] OR sao tome and principe[MeSH] OR saudi arabia[MeSH] OR senegal[MeSH] OR serbia[MeSH] OR seychelles[MeSH] OR sierra leone[MeSH] OR slovakia[MeSH] OR slovenia[MeSH] OR melanesia[MeSH] OR somalia[MeSH] OR south africa[MeSH] OR south sudan[MeSH] OR sri lanka[MeSH] OR saint kitts and nevis[MeSH] OR saint lucia[MeSH] OR saint vincent and the grenadines[MeSH] OR sudan[MeSH] OR suriname[MeSH] OR syria[MeSH] OR tajikistan[MeSH] OR tanzania[MeSH] OR thailand[MeSH] OR timor leste[MeSH] OR togo[MeSH] OR tonga[MeSH] OR trinidad and tobago[MeSH] OR tunisia[MeSH] OR turkey[MeSH] OR turkmenistan[MeSH] OR uganda[MeSH] OR ukraine[MeSH] OR uruguay[MeSH] OR uzbekistan[MeSH] OR vanuatu[MeSH] OR venezuela[MeSH] OR vietnam[MeSH] OR middle east[MeSH] OR yemen[MeSH] OR yugoslavia[MeSH] OR zambia[MeSH] OR zimbabwe[MeSH] OR africa south of the sahara[MeSH] OR africa, central[MeSH] OR africa, northern[MeSH] OR africa, southern[MeSH] OR africa, eastern[MeSH] OR africa, western[MeSH] OR west indies[MeSH] OR indian ocean islands[MeSH] OR caribbean region[MeSH] OR central america[MeSH] OR latin america[MeSH] OR south america[MeSH] OR asia, central[MeSH] OR asia, northern[MeSH] OR asia, southeastern[MeSH] OR asia, western[MeSH] OR europe, eastern[MeSH] OR developing countries[MeSH])
