# Supplementary material for: Evidence for an expanded hypertension care cascade in low- and middle-income countries: a scoping review
Source: BMC Health Serv Res. 2022 Jun 27;22:827. doi: 10.1186/s12913-022-08190-0 (PMC9235242; doi:10.1186/s12913-022-08190-0)
Supplement: Supplementary file 3 — Additional file 3: Quality assessment for studies included in framework development [file 12913_2022_8190_MOESM3_ESM.pdf]

Annex 3: Quality Assessment for Studies Included in Framework Development

There is growing consensus that quality assessment is necessary for observational studies included in systematic review, yet there is disagreement about the method used to assess quality (Mallen, Peat, and Croft 2006; Sanderson, Tatt, and Higgins 2007; Lang and Kleijnen 2010). One major divergence in the current tools available for assessing quality in observational studies is the difference in tools designed to assess the quality of *the actual study* and tools designed to assess the quality of *reporting* (Ma et al. 2020). A common example of a tool to assess the quality of reporting in observational studies is the Strengthening the Reporting of Observational Studies in Epidemiology (STROBE) tool, however these guidelines are not meant to function as a quality assessment for studies (von Elm et al. 2007). Evaluating the quality of the underlying study is preferable in the context of translating findings into the development of a new framework. Two commonly used tools to assess quality of observational studies include the Newcastle-Ottawa Scale (NOS) for assessing the quality of nonrandomized studies in meta-analysis and the Appraisal tool for Cross-Sectional Studies (AXIS tool). Previous researchers could not find materials describing the development process for the NOS tool published in peer-reviewed journals (Stang 2010). On the other hand, the AXIS tool was developed with a systematic Delphi process, which was documented and published in a peer-reviewed journal (Downes et al. 2016). Therefore, this quality assessment relies on the AXIS tool to determine the quality of the studies which inform the development of a framework for measuring the cascade of hypertension management services. Additionally, the quality assessment compiles the scores on the AXIS tool and provides justification for why each study was included in the framework development.

Table 1: AXIS Quality Assessment for Introduction and Methods Sections

	**Introduction**	**Methods**									
**Study**	Were aims and objectives clear?	Was study design appropriate for stated aims?	Was sample size justified?	Was target population clearly defined?	Was sample frame taken from an appropriate population base?	Was selection process likely to select subjects that were representative of the target population?	Were measures taken to address and categorize non-responders?	Were the risk factor and outcome variables measured appropriate to the aims of the study?	Were the risk factor and outcome variables measured correctly using instruments/measurements that had been trialled, piloted, or previously published?	Is it clear what was used to determine statistical significance and/or precision estimates?	Were methods (including statistical methods) sufficiently described to enable them to be repeated?
**Agudelo (2019)**	Y	Y	Y	Y	Y	N (health areas purposively selected, but subunits and patients randomly selected)	Y	Y	Y	Y	Y
**Arrendondo (2018)**	Y	Y	N (used existing state-level data)	N	N	N	N	Y	Y	N/A	N/A
**Bhandari (2015)**	Y	Y	Y	Y	Y	Y	Y	Y	N (outcome of blood pressure was adequately measured but adherence was measured through self-report)	Y	Y
**Charoendee (2018)**	Y	Y	N	Y	Y	Y	N/A (analysis was aggregated to the province level)	Y	Y	N	N
**Chukwuma (2019)**	Y	Y	N	Y	N	N	N	Y	Y	N/A	Y
**Gabert (2017)**	Y	Y	N	Y	Y	Y	N	Y	Y (provided tools for household survey, process for bp measurement was not well described)	N/A	Y
**Galson (2017)**	Y	Y	Y	Y	Y	Y	Y	Y	Y	Y	Y
**Heller (2020)**	Y	Y	N (it was justified in other referenced studies)	Y	Y	Y	Y (non-response was treated as a result)	Y	Y	Y	Y

	Introduction	Methods									
**Study**	Were aims and objectives clear?	Was study design appropriate for stated aims?	Was sample size justified?	Was target population clearly defined?	Was sample frame taken from an appropriate population base?	Was selection process likely to select subjects that were representative of the target population?	Were measures taken to address and categorize non-responders?	Were the risk factor and outcome variables measured appropriate to the aims of the study?	Were the risk factor and outcome variables measured correctly using instruments/measurements that had been trialled, piloted, or previously published?	Is it clear what was used to determine statistical significance and/or precision estimates?	Were methods (including statistical methods) sufficiently described to enable them to be repeated?
**Jayanna (2019)**	Y	Y	Y	Y	Y	Y	N	Y	Y	Y	N
**Khanam (2014)**	Y	Y	N	Y	Y	Y	N	Y	N (no description of items used to measure adherence)	Y	Y
**Leslie (2019)**	Y	Y	Y (with theory rather than calculation)	Y	Y	Y	N	Y	Y	Y	Y
**Liu (2008)**	Y	Y	N	Y	Y	Y	N	Y	Y	Y	Y
**Lozano (2006)**	Y	Y	N	Y	N/A (all of the sampling information was on a supplemental webpage which no longer is functional)	N/A	N/A	N/A	N/A	Y	Y
**Mackino (2018)**	Y	Y	Y	Y	Y	Y	Y	Y	Y	Y	Y
**Thorogood (2007)**	Y	Y	N	Y	Y	Y	Y	Y	Y	Y	Y
**Wollum (2018)**	Y	Y	N	Y	N	N (population data was from DHS but the facility survey was convenience sample)	Y (in the facility survey)	Y	Y (IHME tool was used)	Y	N
**Zack (2016)**	Y	Y	Y (used entire census)	Y	Y	Y	Y	Y	Y	Y	Y
**Zhao (2020)**	Y	Y	Y	Y	Y	Y	N	Y	Y	Y	Y

Table 2: AXIS Quality Assessment for Results, Discussion, and Other Sections

	Results					Discussion		Other		Judgement	
**Study**	Were the basic data adequately described?	Is there an acceptable non-response rate (<20%)*	Was information about non-responders described?	Were results internally consistent?	Were results presented for all analyses described in methods?	Were the authors' discussions and conclusions justified by the results?	Were the limitations of the study discussed?	Are authors' interpretations of results independent of funding declarations or conflicts of interest?*	Was ethical approval or consent of participants attained?	Overall AXIS score (out of 20)	Why was this study included in the final framework?
**Agudelo (2019)**	Y	Y	Y	Y	Y	Y	Y	Y	Y	19	High quality study which provided information about process quality (type of treatment and adherence)
**Arredondo (2018)**	N	N/A	N	Y	Y	Y	Y	Y	N/A	9	Not an observational study, but a discussion piece which defines effective coverage for hypertension management services
**Bhandari (2015)**	Y	Y	Y	Y	Y	Y	Y	Y	Y	19	High quality study which provided information about process quality (type of treatment and adherence)
**Charoendee (2018)**	Y	N/A	N/A	Y	Y	Y	Y	Y	Y	14	Defines effective coverage of hypertension screening services, which supports the need for an expanded care cascade
**Chukwuma (2019)**	Y	N/A	N/A	Y	Y	Y	Y	Y	Y	13	Uses a hypertension care cascade with supply- and demand-side factors (including structural quality) to explain service coverage
**Gabert (2017)**	Y	N	N	N (text did not match figures)	Y	Y	Y	Y	Y	14	Uses a hypertension care cascade with supply- and demand-side factors (including structural quality) to explain service coverage
**Galson (2017)**	Y	Y (household 15%, individual 20.6%)	Y	Y	Y	Y	Y	Y	Y	20	High quality study which provided information about process quality (type of treatment)
**Heller (2020)**	Y	Y	Y	Y	Y	Y	Y	Y	Y	19	High quality study which documents process quality (referral) throughout the hypertension care cascade (screening, treatment, control)

	Results					Discussion		Other		Judgement	
**Study**	Were the basic data adequately described?	Is there an acceptable non-response rate (<20%)*	Was information about non-responders described?	Were results internally consistent?	Were results presented for all analyses described in methods?	Were the authors' discussions and conclusions justified by the results?	Were the limitations of the study discussed?	Are authors' interpretations of results independent of funding declarations or conflicts of interest?*	Was ethical approval or consent of participants attained?	Overall AXIS score (out of 20)	Why was this study included in the final framework?
**Jayanna (2019)**	Y	Y	N	Y	Y	Y	Y	Y	Y	17	High quality study which describes barriers to process and structural quality
**Khanam (2014)**	Y	N/A	N	Y	Y	Y	Y	Y	Y	15	Describes process quality (adherence to treatment)
**Leslie (2019)**	Y	N/A	N	Y	Y	Y	Y	Y	Y	17	High quality study which defines effective coverage for hypertension management services and multiple measures of outcome quality (non-hospitalization and bp control)
**Liu (2008)**	Y	N/A	N	Y	Y	Y	Y	Y	Y	16	High quality study which defines effective coverage for hypertension management services
**Lozano (2006)**	Y	N/A	N	Y	Y	Y	Y	Y	Y	12	Process documentation for measuring effective coverage which clearly defines a measure for hypertension management services
**Mackino (2018)**	Y	Y	N	Y	Y	Y	N	Y	Y	18	High quality study which describes process quality (obtaining quality care) on a hypertension care cascade
**Thorogood (2007)**	Y	N (non-response of 23%)	N	Y	Y	Y	N	Y	Y	16	High quality study which describes barriers related to structural quality on hypertension care cascade
**Wollum (2018)**	Y	N/A	Y	Y	Y	Y	Y	Y	Y	15	Describes process quality and supply-side barriers to hypertension care
**Zack (2016)**	Y	N (non-response rate of 53%)	Y	Y	Y	Y	Y	Y	Y	19	High quality study which describes process quality (patient adherence)
**Zhao (2020)**	Y	Y	N	Y	Y	Y	Y	Y	Y	19	High quality study which defines effective coverage for hypertension management services

**Indicates questions that were revised from original AXIS tool so that affirmative answers were positively interpreted*

Works Cited

Downes, Martin J., Marnie L. Brennan, Hywel C. Williams, and Rachel S. Dean. 2016. "Development of a Critical Appraisal Tool to Assess the Quality of Cross-Sectional Studies (AXIS)." *BMJ Open* 6 (12): e011458. https://doi.org/10.1136/bmjopen-2016-011458.

Elm, Erik von, Douglas G. Altman, Matthias Egger, Stuart J. Pocock, Peter C. Gøtzsche, and Jan P. Vandenbroucke. 2007. "The Strengthening the Reporting of Observational Studies in Epidemiology (STROBE) Statement: Guidelines for Reporting Observational Studies." *Lancet* 370 (9596): 1453–57. https://doi.org/10.1016/S0140-6736(07)61602-X.

Lang, Shona, and Jos Kleijnen. 2010. "Quality Assessment Tools for Observational Studies." *International Journal of Evidence-Based Healthcare* 8 (4): 247. https://doi.org/10.1111/j.1744-1609.2010.00195.x.

Ma, Lin Lu, Yun Yun Wang, Zhi Hua Yang, Di Huang, Hong Weng, and Xian Tao Zeng. 2020. "Methodological Quality (Risk of Bias) Assessment Tools for Primary and Secondary Medical Studies: What Are They and Which Is Better?" *Military Medical Research*. BioMed Central Ltd. https://doi.org/10.1186/s40779-020-00238-8.

Mallen, Christian, George Peat, and Peter Croft. 2006. "Quality Assessment of Observational Studies Is Not Commonplace in Systematic Reviews." *Journal of Clinical Epidemiology* 59 (8): 765–69. https://doi.org/10.1016/j.jclinepi.2005.12.010.

Sanderson, S., I. D Tatt, and J. P. Higgins. 2007. "Tools for Assessing Quality and Susceptibility to Bias in Observational Studies in Epidemiology: A Systematic Review and Annotated Bibliography." *International Journal of Epidemiology* 36 (3): 666–76. https://doi.org/10.1093/ije/dym018.

Stang, Andreas. 2010. "Critical Evaluation of the Newcastle-Ottawa Scale for the Assessment of the Quality of Nonrandomized Studies in Meta-Analyses." *European Journal of Epidemiology*. https://doi.org/10.1007/s10654-010-9491-z.
