# Supplementary material for: Evidence for an expanded hypertension care cascade in low- and middle-income countries: a scoping review
Source: BMC Health Serv Res. 2022 Jun 27;22:827. doi: 10.1186/s12913-022-08190-0 (PMC9235242; doi:10.1186/s12913-022-08190-0)
Supplement: Supplementary file 4 — Additional file 4: World bank reports that incorporate measures of service quality to measures of hypertension service coverage [file 12913_2022_8190_MOESM4_ESM.pdf]

Annex 4: World Bank reports that incorporate service-quality to measures of hypertension management service coverage

Author, year	Study Type/Data source	Study population	Care cascade	Quality Measure Reported	Notes
[73]	Longitudinal<br><br>Cohort study , patient files from clinics	South Africa: 4 Districts (Bojanala, Ekurhuleni, Mopani, and King Cetshwayo)<br><br>Adults 18 and older	Diagnosed<br><br>Initiated treatment<br><br>One or more visits within first 3 months<br><br>Visit or BP measure at 6 months<br><br>Disease control achieved	Visit compliance in the first 3 months<br><br>Initiated on hypertension treatment<br><br>Regimen at initiation<br><br>Retention in care	Also had a hypertension screening cascade
[74]	Cross-sectional<br><br>DHS and SPA data	Bangladesh (national)<br><br>Adults 18 and older	Hypertensive<br><br>Diagnosed<br><br>Treated<br><br>BP controlled	Percent of health facilities providing CVD services<br><br>Percent of facilities with CVD medication available	Reported supply- and demand-side barriers in the continuum of care to screening/diagnosis, treatment initiation, treatment monitoring, and treatment adherence/disease control
[75]	Mixed-methods (Cross-sectional)<br><br>Household survey, patient file data extraction, focus group discussions with patients and care providers	Samoa: two islands (Upolu and Savai'i)<br><br>Adults 20 and older	Hypertensive<br><br>BP screened<br><br>Diagnosed<br><br>Treatment initiated<br><br>BP monitored according to standards<br><br>BP controlled	Availability of medicines and equipment<br><br>Treatment advice from doctors<br><br>Adherence	Reported barriers and facilitators at each stage in the hypertension cascade
